# Relationship between maternal pre-pregnancy BMI and neonatal birth weight in pregnancies with gestational diabetes mellitus: a retrospective cohort study

**DOI:** 10.3389/fmed.2024.1478907

**Published:** 2025-01-06

**Authors:** Qiuping Liao, Tiantian Yu, Jiajia Chen, Xiuqiong Zheng, Lianghui Zheng, Jianying Yan

**Affiliations:** ^1^Fujian Maternity and Child Health Hospital, College of Clinical Medicine for Obstetrics & Gynecology and Pediatrics, Fujian Medical University, Fuzhou, China; ^2^Fujian Clinical Research Center for Maternal-Fetal Medicine, Fuzhou, China; ^3^National Key Obstetric Clinical Specialty Construction Institution of China, Fuzhou, China

**Keywords:** GDM, neonatal birth weight, pre-pregnancy BMI, small for gestational age, large for gestational age

## Abstract

**Aim:**

The aim of this study was to explore the association between maternal pre-pregnancy body mass index (BMI) and neonatal birth weight in pregnancies with gestational diabetes mellitus (GDM).

**Methods:**

This was a retrospective cohort study conducted between January 2019 and June 2020 at a university hospital in Fuzhou, China.

**Results:**

Pre-pregnancy BMI was used to categorize 791 pregnant women as underweight (3.03%), normal weight (51.71%), overweight (32.74%), and obese (12.52%). Among the 791 babies, 11.63% were small for gestational age (SGA), 77.37% were normal weight, and 11.00% were large for gestational age (LGA). The rate of the SGA babies increased with higher pre-pregnancy BMI. The percentage of LGA babies was higher in women who were overweight or obese compared to those of normal weight. Neonatal birth weight displayed a significantly increasing trend with increasing maternal pre-pregnancy BMI when maternal pre-pregnancy BMI was less than 27.78 kg/m^2^ [*β* = 0.03, 95% CI (0.01, 0.04); *p* = 0.0052 < 0.05] when maternal pre-pregnancy BMI was greater than 27.78 kg/m^2^, neonatal birth weight decreased as maternal pre-pregnancy BMI increased [*β* = −0.01, 95% CI (−0.04, 0.01); *p* = 0.3555].

**Conclusion:**

The incidence of SGA and LGA babies was higher in the women with GDM who were overweight or obese before pregnancy. The data suggest that different management strategies should be implemented for pregnant women with a pre-pregnancy BMI below 27.78 kg/m^2^ and above 27.78 kg/m^2^, particularly in cases of GDM. These findings highlight the importance of providing information, offering preconception counseling, and delivering health education on weight management to ensure healthy pregnancies.

## 1 Introduction

Gestational diabetes mellitus (GDM) is a metabolic disorder that occurs during pregnancy and can lead to adverse fetal outcomes, as well as maternal and offspring complications (1). The hyperglycemia and adverse pregnancy outcome study has confirmed a linear association between pregnancy complications and maternal glycemia (2, 3). The global prevalence of gestational diabetes is estimated to be 14.0% according to the International Association of the Diabetes and Pregnancy Study Groups (IADPSG) criteria (4). In China, the prevalence of GDM has sharply increased over the past decade from 4% in 2010 to 21% in 2020 (5). Adverse maternal or offspring outcomes are common in women with GDM and can vary based on the underlying metabolic conditions (1). Identifying strategies to mitigate pregnancy risks in this group of women is essential.

A common and potentially modifiable risk factor associated with adverse fetal and neonatal outcomes during pregnancy is maternal obesity. The incidence of obesity has markedly increased worldwide, with global prevalence rates of 23.0% for overweight and 16.3% for obesity, respectively (6). Obese women of reproductive age represent a clinically important subpopulation. It is well known that obesity is linked to complications commonly associated with GDM, such as hypertensive disorders of pregnancy (HDP), gestational proteinuria, postpartum hemorrhage, preterm delivery, fetal malformation or stillbirth, neonatal asphyxia, large for gestational age (LGA), shoulder dystocia, and higher rates of cesarean section (7). Regarding the relationship between maternal pre-pregnancy body mass index (BMI) and neonatal birth weight, recent studies have presented different perspectives. Some researchers have reported a positive correlation, while others have argued that there is no correlation (8, 9).

The relationship between maternal pre-pregnancy BMI and neonatal birth weight remains debatable, especially in cases of GDM. Understanding the impact of maternal pre-pregnancy weight on neonatal outcomes in cases of GDM may enhance pre-conception counseling and help develop strategies to improve adverse outcomes. Therefore, we sought to examine the specific relationship between maternal pre-pregnancy weight and neonatal birth weight in cases of GDM.

## 2 Methods

### 2.1 Data sources

This study adhered to the principles outlined in the Declaration of Helsinki and was approved by the Ethics Committee of Fujian Maternity and Child Health Hospital, Fuzhou, Fujian, China (2023KY046). A retrospective cohort study was conducted at the Department of Obstetrics at Fujian Maternity and Child Health Hospital, Fuzhou, China from January 2019 to June 2020. A total of 797 pregnant women with GDM were enrolled in this study. Data were collected from the medical history of the participants. In the present study, subjects were selected based on the GDM screening criteria of the World Health Organization (WHO). All pregnant women between 24 and 28 gestational weeks underwent an oral glucose tolerance test (OGTT) with a 75 g glucose load. Pregnancy with any of the glucose values at or above the specific thresholds (≥5.1 mmol/L for fasting, ≥10 mmol/L at 1 h, ≥8.5 mmol/L at 2 h) was defined as GDM (10). Pregnant women with pre-existing diabetes mellitus or multiple pregnancies were excluded. A total of 791 matched pairs of maternal pre-pregnancy BMI and neonatal birth weight were analyzed, due to information missing for 6 pairs.

### 2.2 Outcome measures

Maternal characteristics extracted from medical records included age, nationality, education level, history of any abnormal pregnancy, pre-pregnancy BMI, and neonatal birth weight. Pre-pregnancy BMI was calculated by dividing weight in kilograms by the square of height in meters (kg/m^2^). According to the guidelines issued by the Institute of Medicine (IOM) in 2009, participants were divided into four groups based on their pre-pregnancy BMI: underweight (BMI <18.5 kg/m^2^), normal weight (BMI 18.5 to 24.9 kg/m^2^), overweight (BMI 25 to 29.9 kg/m^2^), and obese (BMI ≥30 kg/m^2^) (11).

Large-for-gestational-age (LGA) infants are defined as those with a birth weight ≥the 90th percentile for gestational age, typically weighing ≥4 kg. Small-for-gestational-age [SGA, also known as fetal growth restriction (FGR)] infants are defined as those with a birth weight below two standard deviations from the mean weight for the same gestational age or below the 10th percentile of normal weight for the same gestational age (12).

### 2.3 Statistical analysis

Univariate and multivariate piecewise linear regression analyses were performed using the statistical software packages R (http://www.R-project.org, The R Foundation) and EmpowerStats (http://www.empowerstats.com, X&Y Solutions, Inc., Boston, MA). Column graphs were created using GraphPad Prism 5 (GraphPad Software). Continuous variables with a normal distribution were expressed as mean (standard deviation). Categorical variables were expressed as frequencies or percentages. A univariate analysis model was used to determine the significance of the association between maternal pre-pregnancy BMI and neonatal birth weight, along with other independent variables. A multivariate regression model was further used to examine the independent association between maternal pre-pregnancy BMI and neonatal birth weight. The relationship between maternal pre-pregnancy BMI and neonatal birth weight was explored using smooth curve fitting after adjusting for potential confounders. Statistical significance (*p* < 0.05) was determined using the *t*-test.

## 3 Results

### 3.1 Baseline characteristics of all participants

The baseline characteristics of participants are described in Table 1. A total of 797 patients with GDM were included in the present study. Their mean age was 31.50 ± 5.30 years. Of the 797 patients, 790 (99.12%) were ethnic Han. The frequencies of GDM with a family history of diabetes, hypertension, and abnormal pregnancy history were 14.81, 2.63, and 18.70%, respectively. Male infants accounted for 52.57%. Approximately 77.42% of newborns were born by cesarean section.

**Table 1:** Baseline characteristics of participants.

Characteristics	Mean ± SD/*n* (%)
Maternal age, years	31.50 ± 5.30
Maternal pre-pregnancy BMI, kg/m^2^	
<18.5	24 (3.03%)
≥18.5, <24.9	409 (51.71%)
≥25, <29.9	259 (32.74%)
≥30	99 (12.52%)
Birth weight, kg	
<2.5	92 (11.63%)
≥2.5, <4.0	612 (77.37%)
≥4.0	87 (11.00%)
Nationality	
Ethnic Han	790 (99.12%)
Other	7 (0.88%)
Family history of diabetes	118 (14.81%)
Pre-pregnancy hypertension	21 (2.63%)
Delivery mode	
Caesarean section	617 (77.42%)
Vaginal delivery	180 (22.58%)
Infant sex	
Male	419 (52.57%)
Female	378 (47.43%)
Abnormal pregnancy history	149 (18.70%)

SD, standard deviation; BMI, body mass index.

**Figure 1:**
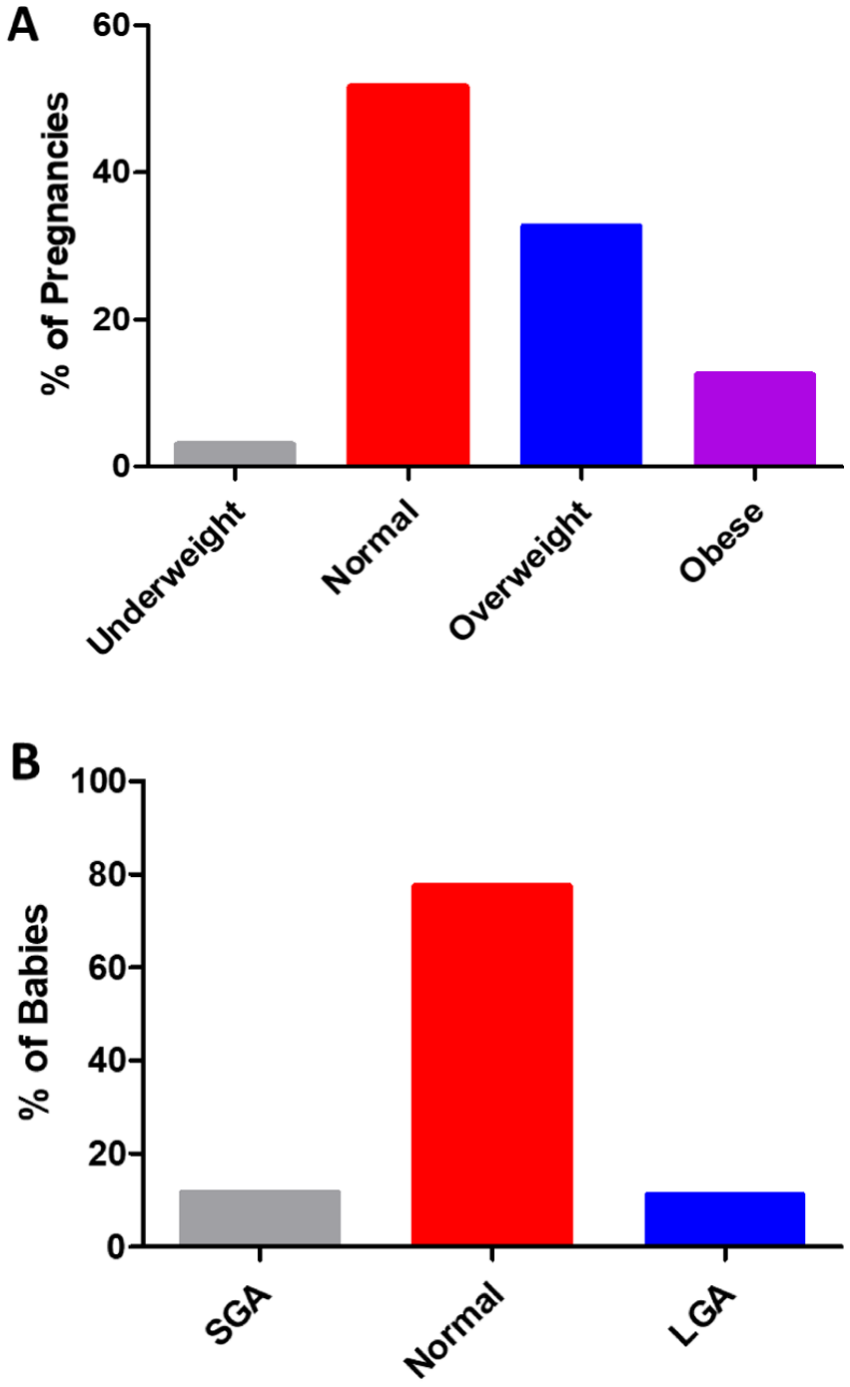
**(A)** Of the 791 pregnancies in the women with GDM, 3.03% occurred in those who were underweight (BMI <18.5 kg/m^2^), 51.71% in those with normal weight (BMI 18.5 to 24.9 kg/m^2^), 32.74% in those who were overweight (BMI 25 to 29.9 kg/m^2^), and 12.52% in those with obesity (BMI ≥30 kg/m^2^). **(B)** The distribution of birth weight in the total of 791 babies from the GDM pregnancies. A total of 11.63% were SGA (<2.5 kg), 77.37% were normal weight (≥2.5, <4.0 kg), and 11.00% were LGA (≥4.0 kg).

**Figure 2:**
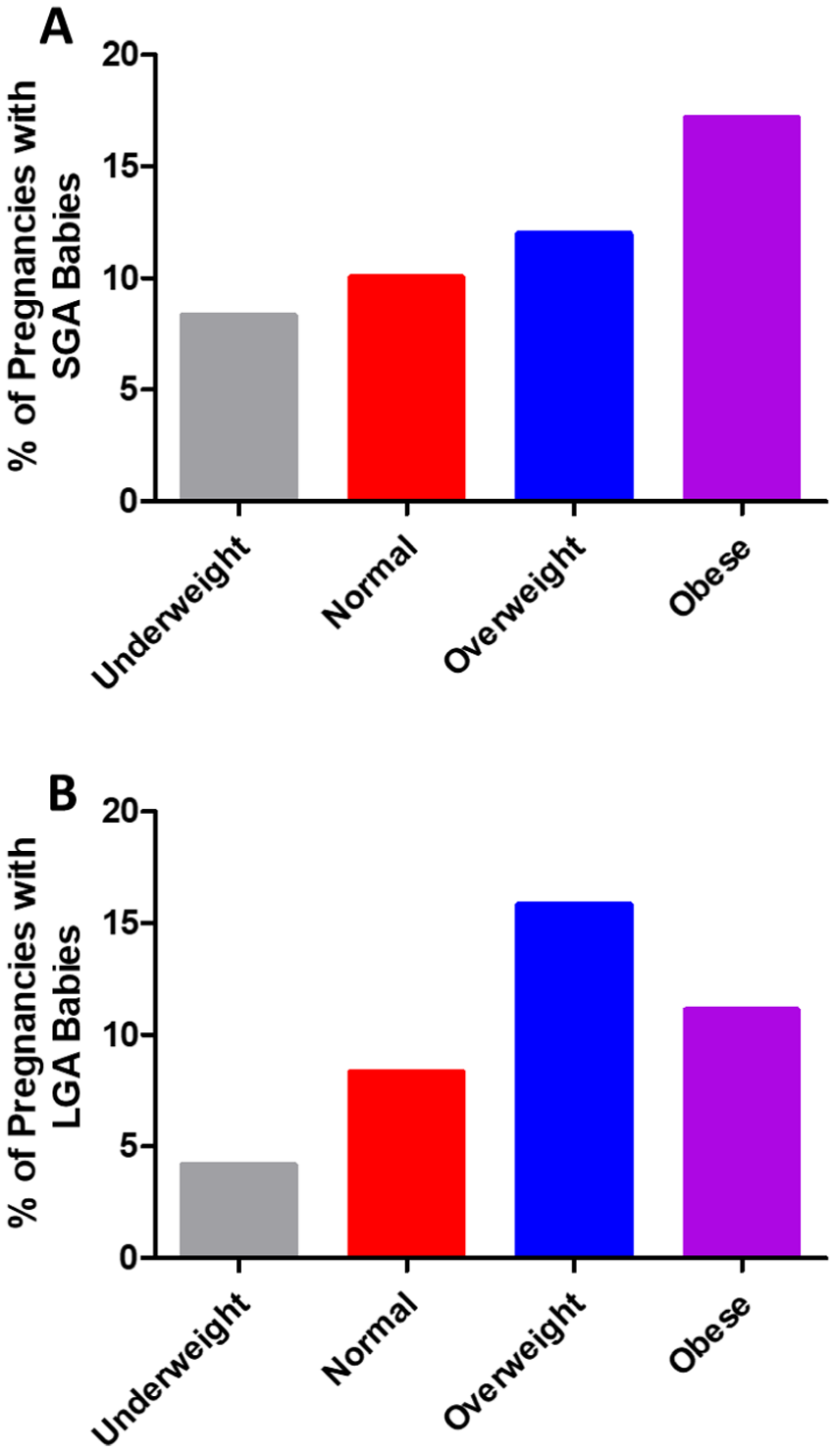
**(A)** Small for gestational age (SGA) and **(B)** large for gestational age (LGA) babies were more common in the pregnancies of the women who were overweight or obese compared with those who were underweight or of normal weight in the cases of GDM.

In 791 pregnancies with GDM, 12.52% of the pregnancies occurred in women with obesity, 32.74% in overweight women, 51.71% in women with normal weight, and 3.03% in underweight women (Figure 1A). As shown in Figure 1B, the weight of 791 babies was analyzed. In the present study, 77.37% of the babies had normal weight, 11.63% were classified as SGA, and 11.00% as LGA. The number of SGA babies rose steadily as pre-pregnancy BMI increased (Figure 2A). In addition, the percentage of LGA babies was higher in the women who were overweight and obese compared to normal-weight women (Figure 2B).

### 3.2 Factors associated with neonatal birth weight

Univariate linear regression analysis was performed to determine the relationship between the clinical parameters and neonatal birth weight. As shown in Table 2, in the unadjusted model, we observed no significant association between maternal pre-pregnancy BMI and neonatal birth weight (*p* > 0.05). Moreover, no significant association was observed between neonatal birth weight and a family history of diabetes or mode of delivery (*p* > 0.05). Notably, there was a significant negative relationship between neonatal birth weight and maternal age [*β* = −0.01, 95% CI (−0.02, −0.00); *p* = 0.045], nationality [*β* = −0.88, 95% CI (−1.39, −0.37); *p* = 0.001], pre-pregnancy hypertension [*β* = −0.89, 95% CI (−1.18, −0.60); *p* < 0.000], infant sex [*β* = −0.11, 95% CI (−0.20, −0.01); *p* = 0.029], and abnormal pregnancy history [*β* = −0.19, 95% CI (−0.31, −0.07); *p* = 0.003].

**Table 2:** Factors correlated to neonatal birth weight by a univariate analysis.

Covariate	*β* (95% CI)	*p*-value
Maternal age, years	−0.01 (−0.02, −0.00)	0.045^*^
Maternal pre-pregnancy BMI, kg/m^2^	0.01 (−0.01, 0.02)	0.279
Nationality, *n* (%)		
Ethnic Han	Reference	
Other	−0.88 (−1.39, −0.37)	0.001^***^
Family history of diabetes, *n* (%)	0.02 (−0.12, 0.16)	0.772
Pre-pregnancy hypertension, *n* (%)	−0.89 (−1.18, −0.60)	<0.000^***^
Delivery mode, *n* (%)		
Caesarean section	Reference	
Vaginal delivery	0.01 (−0.11, 0.12)	0.873
Infant sex, *n* (%)		
Male	Reference	
Female	−0.11 (−0.20, −0.01)	0.029^*^
Abnormal pregnancy history, *n* (%)	−0.19 (−0.31, −0.07)	0.003^**^

SD: standard deviation; BMI: body mass index. Statistically significant: ^*^p < 0.05, ^**^p < 0.01, and ^***^p < 0.001.

### 3.3 Relationship between maternal pre-pregnancy BMI and neonatal birth weight

**Figure 3:**
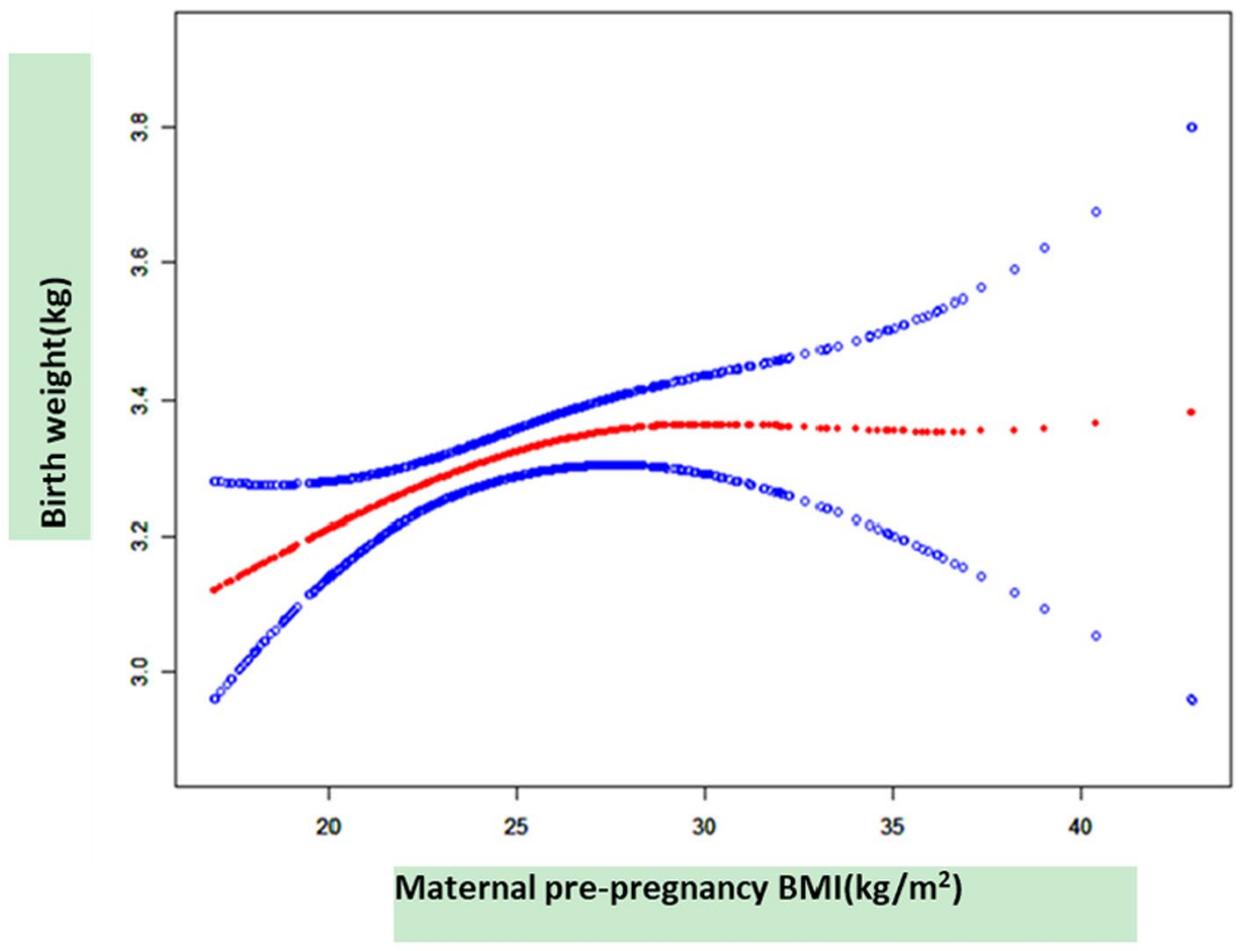
The relationship between maternal pre-pregnancy BMI and neonatal birth weight, as determined by smooth curve fitting.

As shown in Figure 3, smooth curve fitting was performed after adjusting for possible factors, including maternal age, status, nationality, a family history of diabetes, abnormal pregnancy history, infant sex, and mode of delivery. Maternal pre-pregnancy BMI exhibited a non-linear relationship with neonatal birth weight, and the resulting curve exhibited a two-stage change with a breakpoint at 27.78 kg/m^2^. When the maternal pre-pregnancy BMI value was below the breakpoint, there was a positive relationship between maternal pre-pregnancy BMI and neonatal birth weight. However, when the value exceeded the breakpoint, there was an inverse relationship between maternal pre-pregnancy BMI and neonatal birth weight. Table 3 shows that the threshold effect was further analyzed based on curve fitting. Specifically, neonatal birth weight displayed a significantly increasing trend with increasing maternal pre-pregnancy BMI when maternal pre-pregnancy BMI was less than 27.78 kg/m^2^ [*β* = 0.03, 95%CI (0.01, 0.04); *p* = 0.0052 < 0.05]. However, when maternal pre-pregnancy BMI was greater than 27.78 kg/m^2^, neonatal birth weight decreased as maternal pre-pregnancy BMI increased [*β* = −0.01, 95% CI (−0.04, 0.01); *p* = 0.3555].

**Table 3:** The independent association between maternal pre-pregnancy BMI (kg/m^2^) and neonatal birth weight (kg) by multivariate piecewise linear regression.

Maternal pre-pregnancy BMI	*β* (95% CI)	*p*-value
<27.78	0.03 (0.01, 0.04)	0.005^**^
≥27.78	−0.01 (−0.04, 0.010)	0.356

BMI, body mass index. Effect: neonatal birth weight. Cause: maternal pre-pregnancy BMI. adjusted: pre-pregnancy hypertension, maternal age, nationality, family history of diabetes, abnormal pregnancy history, infant sex, delivery mode. Statistically significant: ^**^p < 0.01.

## 4 Discussion

In this retrospective cohort study, approximately half of the women with GDM were overweight or obese before pregnancy. In contrast, very few women were underweight. The women who were overweight or obese before pregnancy were more likely to have SGA or LGA births, especially in the cases of GDM. Furthermore, we observed a non-linear relationship between maternal pre-pregnancy BMI and neonatal birth weight in cases of GDM, with the turning point of maternal pre-pregnancy BMI at 27.78 kg/m^2^. When the maternal pre-pregnancy BMI value was below the breakpoint, there was a positive relationship between maternal pre-pregnancy BMI and neonatal birth weight. However, when the value exceeded the breakpoint, the relationship reversed. The data suggest that different management strategies should be implemented for pregnant women with a pre-pregnancy BMI value below 27.78 kg/m^2^ and above 27.78 kg/m^2^, particularly in cases of GDM. These findings highlight the importance of providing information, preconception counseling, and health education on weight management for healthy pregnancies.

Previous studies have shown that the fetuses of pregnant women with GDM may be macrosomic, SGA, or of normal birth weight, depending on the severity of diabetes and the degree of diabetes control (2, 13). Better control of diabetes normalizes fetal growth, while severe diabetes often results in SGA fetuses (14). Although there are different causes for FGR or LGA, paradoxically, both are related to the “metabolic syndrome” (15). Therefore, we are working to find a way to improve maternal-fetal outcomes before pregnancy.

In a previous study, it was confirmed that there is a direct relationship between maternal BMI and neonatal birth weight (16). Another study supported the view that pre-pregnancy BMI is positively associated with the birth weight of neonates in cases of GDM (17). However, some researchers have pointed out that pre-gestational BMI is not significantly associated with macrosomia (18). Such discrepancies may be attributed to other factors, such as gestational diabetes treatment during pregnancy, pathological obesity, and ethnic differences.

In previous studies, maternal age, family history of diabetes, education, week of gestation at diagnosis, and neonatal sex were the adjusted variables that affected the relationship between maternal pre-pregnancy BMI and neonatal birth weight (17, 19). In our study, we enrolled women who were diagnosed with GDM between 24 and 28 gestational weeks, and we excluded participants with multiple pregnancies and those with pre-pregnancy diabetes mellitus. In addition, we adjusted for maternal family history of diabetes and abnormal pregnancy history. The information obtained from our study showed that maternal pre-pregnancy overweight/obesity evidently increased the rates of SGA or LGA births. It is worth noting that a non-linear relationship was observed between maternal pre-pregnancy BMI and neonatal birth weight. When the maternal pre-pregnancy BMI value was ≤27.78 kg/m^2^, the relationship was positive. However, when the maternal pre-pregnancy BMI value was ≥27.78 kg/m^2^, the relationship was reversed.

Pre-conception counseling should be offered to all women with GDM. The pre-conception assessment should be performed by an endocrinologist or obstetrician with expertise in diabetes and pregnancy. This evaluation should address pregnancy risks, medication safety, and the necessary interventions to optimize pre-pregnancy health. For women who are overweight or obese, it is recommended to review lifestyle modifications for weight reduction, provide guidance on appropriate weight gain during pregnancy, and screen for comorbidities. Medical nutritional therapy and exercise have been proven to be effective in lowering LGA and macrosomia rates without increasing SGA rates (20). A meta-analysis of randomized trials focusing on lifestyle interventions during pregnancy showed that improving BMI before pregnancy, rather than during pregnancy, may be more effective in the prevention of pregnancy or offspring complications (21). Based on this study, women should also be educated about the association between maternal pre-pregnancy weight and adverse newborn outcomes.

Neonatal birth weight and pre-pregnancy BMI values were obtained from the hospital’s medical record system, which reduced potential bias in the study. Moreover, the data included specific maternal information, which was helpful in adjusting for potential confounding factors. In addition, information on maternal age at conception, mode of delivery, and fetal sex was available and helped us to control for confounding factors.

Inevitably, our study has several limitations. First, this was a retrospective study, and thus, the collected data may have introduced bias into the results of this study. Second, the specimens were obtained from only one district. Therefore, the results of the study are not nationally representative and may not apply to all women in China or to populations beyond this region. Finally, this study lacked a control group. Setting a control parameter would allow us to visualize fetal birth weight changes with pre-pregnancy BMI changes in cases without gestational diabetes.

Overall, the association between pre-pregnancy BMI and fetal birth weight should be further investigated in other populations to confirm our results. Establishing a clear relationship between pre-pregnancy body mass index and fetal birth weight is crucial for developing early prevention strategies for pregnant women with gestational diabetes.

## 5 Conclusion

We described a non-linear relationship between maternal pre-pregnancy BMI and neonatal birth weight in the women with GDM, after adjusting for potential confounders. The turning point of maternal pre-pregnancy BMI was 27.78 kg/m^2^. Neonatal birth weight displayed a significantly increasing trend with increasing maternal pre-pregnancy BMI when maternal pre-pregnancy BMI was less than 27.78 kg/m^2^. However, when maternal pre-pregnancy BMI was greater than 27.78 kg/m^2^, neonatal birth weight decreased as maternal pre-pregnancy BMI increased. These findings highlight the importance of providing information, preconception counseling, and health education on weight management for healthy pregnancies.

## Data Availability

The raw data supporting the conclusions of this article will be made available by the authors, without undue reservation.
